# Genetic Screen for Regulators of Lymph Gland Homeostasis and Hemocyte Maturation in *Drosophila*

**DOI:** 10.1534/g3.111.001693

**Published:** 2012-03-01

**Authors:** Kai Li Tan, Siow Chong Goh, Svetlana Minakhina

**Affiliations:** Waksman Institute, Rutgers University, Piscataway, New Jersey 08854

**Keywords:** lymph gland, *Drosophila*, hematopoiesis, Zfrp8

## Abstract

Blood cell development in the *Drosophila* lymph gland is controlled by multiple factors, most of them conserved from flies to mammals. The *Drosophila* homolog of vertebrate PDCD2, Zfrp8, is required in *Drosophila* hematopoietic stem cell development. *Zfrp8* mutant larvae show a disruption of homeostasis in the lymph gland and vast lymph gland overgrowth. The loss of one copy of *Zfrp8* also causes a lymph gland enlargement. This dominant phenotype can be modified by heterozygous mutations in cell-cycle genes and several genes functioning in blood development. To identify additional genes that function in hematopoiesis, we screened a collection of second and third chromosome deficiencies for modifiers of *Zfrp8* heterozygous phenotype. Using deficiency mapping, available single gene mutations, and RNAi lines, we identified several novel factors required for lymph gland development and hemocyte differentiation. Distinct lymph gland phenotypes of nine of these genes are reported here for the first time. Importantly, the orthologs of four of them have a role in mammalian blood development and leukemogenesis. Our work has shown that the number of genes regulating normal blood cell development in *Drosophila* is much larger than expected, and that the complex molecular mechanisms regulating hemocyte differentiation are comparable to those in vertebrates.

In vertebrates, hematopoietic stem cells (HSC) give rise to at least eight hematopoietic lineages. Studies of signaling pathways and transcription factors revealed a number of factors controlling decision points in hematopoiesis. The differentiation along each lineage is orchestrated by transcription regulators, including PU.1, Foxo1, EBF1, Runx, and GATA factors. Notch, JAK/STAT, and Wingless (Wg) signaling are implicated in regulation of self-renewal [reviewed in Mercer *et al.* (2011) and Orkin and Zon (2008)]. Modern genomic approaches uncovered many more genes expressed during blood cell differentiation and identified regulatory networks involving hundreds of transcription factors (Novershtern *et al.* 2011). Although the role of transcriptional control in hematopoiesis cannot be underestimated, pre- and posttranscriptional mechanisms also play important roles in hematopoiesis, but only few examples have been studied (Baou *et al.* 2011; Sardina *et al.* 2011; Yoshida *et al.* 2011).

Hematopoiesis in *Drosophila* is simpler than in vertebrates and results in the differentiation of three major types of hemocytes: plasmatocytes, crystal cells, and lamellocytes [for review see (Crozatier and Meister 2007; Evans and Banerjee 2003; Lanot *et al.* 2001; Lebestky *et al.* 2000]. Like in humans, the best-characterized genes in *Drosophila* hematopoiesis are transcription factors and genes encoding components of signal transduction pathways. For example the transcription factor lozenge (Lz), a Runx domain protein, is required for crystal cell specification, and the GATA transcription factor pannier (Pnr) is essential for plasmatocyte development (Lebestky *et al.* 2000; Minakhina *et al.* 2011). Jak/Stat signaling controls several processes, including both plasmatocyte and lamellocyte differentiation (Luo *et al.* 2002; Minakhina *et al.* 2011). The Wingless (Wg), Hedgehog (Hh), and Notch pathways also function at several stages of lymph gland development (Mandal *et al.* 2007; Mukherjee *et al.* 2011; Muratoglu *et al.* 2006; Sinenko *et al.* 2009; Tokusumi *et al.* 2010). Recent studies by Avet-Rochex *et al.* (2010) suggest that hemocyte differentiation relies on diverse cellular mechanisms, but genes other than transcription factors are not well studied in vertebrates and invertebrate models.

## *Drosophila* lymph gland structure and development

**Figure 1:**
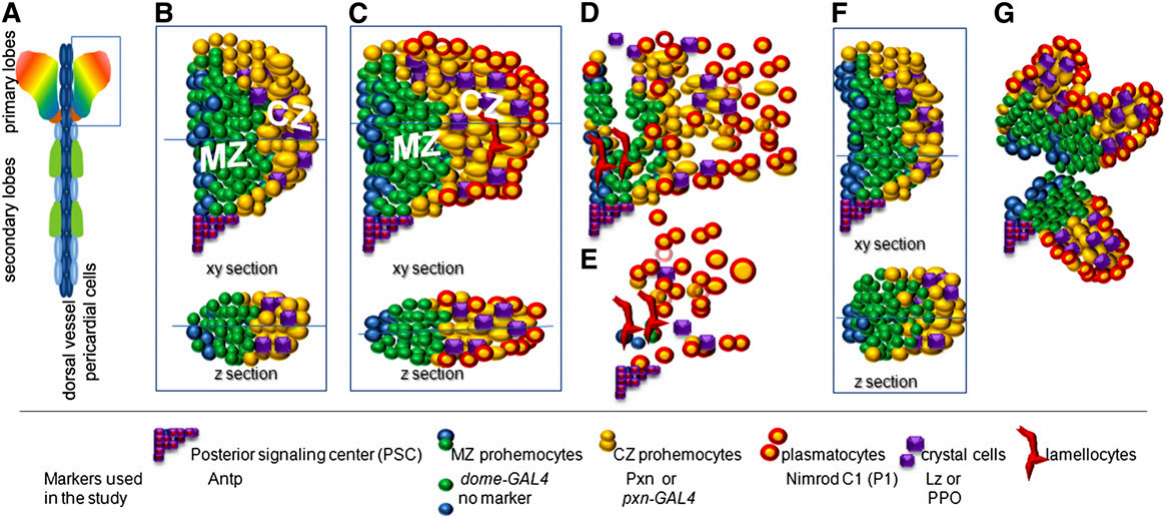
Schematic representation of the lymph gland structure. (A) Whole lymph gland with primary and secondary lobes. (B–C) Cross-sections and z-sections of normal primary lobe of middle (B) and late (C) third-instar larval lymph glands. MZ prohemocytes expressing *dome-GAL4* are shown in green. Prohemocytes expressing no markers are blue. CZ prohemocytes expressing Pxn are shown in yellow, crystal cells are purple, and lamellocytes are elongated red cells. Plasmatocytes (encircled in red) first differentiate at the surface of the CZ. (D, E) Primary lobes disintegrate upon metamorphosis or in response to immune challenge. Disintegration is also observed in a number of mutants as a result of accelerated hemocyte differentiation. (F, G) Examples of abnormally shaped primary lobes observed during the screen. Markers and their expression used in this study are indicated below the drawing.

In *Drosophila*, the lymph gland is the major source of adult hemocytes and has been developed as a model to study hematopoiesis. The lymph gland is established during embryogenesis as paired clusters of cells associated with the cardiovascular system, alongside the dorsal vessel (Jung *et al.* 2005; Mandal *et al.* 2004). Clonal analysis shows that embryonic and first-instar lymph glands contain pluripotent cells with the ability to form cell lineages, reminiscent of vertebrate HSCs (Minakhina and Steward 2010). After the transition to the second-instar larval stage, the pluripotency is no longer observed (Krzemien *et al.* 2010). During the second- and third-instar larval stages, the lymph glands expand and form primary lobes and a series of secondary and tertiary lobes located posterior to the primary lobes alongside the dorsal vessel (Figure 1A). At the third-instar stage, the primary lobe can be subdivided into three structurally distinct compartments: the medullary zone (MZ), with tightly packed cells located proximally to the dorsal vessel; the petal-shaped distal cortical zone (CZ); with larger and more loosely packed cells; and a hematopoietic niche, the posterior signaling center (PSC; Figure 1, B and C) (Jung *et al.* 2005; Krzemien *et al.* 2007; Lebestky *et al.* 2003; Mandal *et al.* 2007).

**Figure 2:**
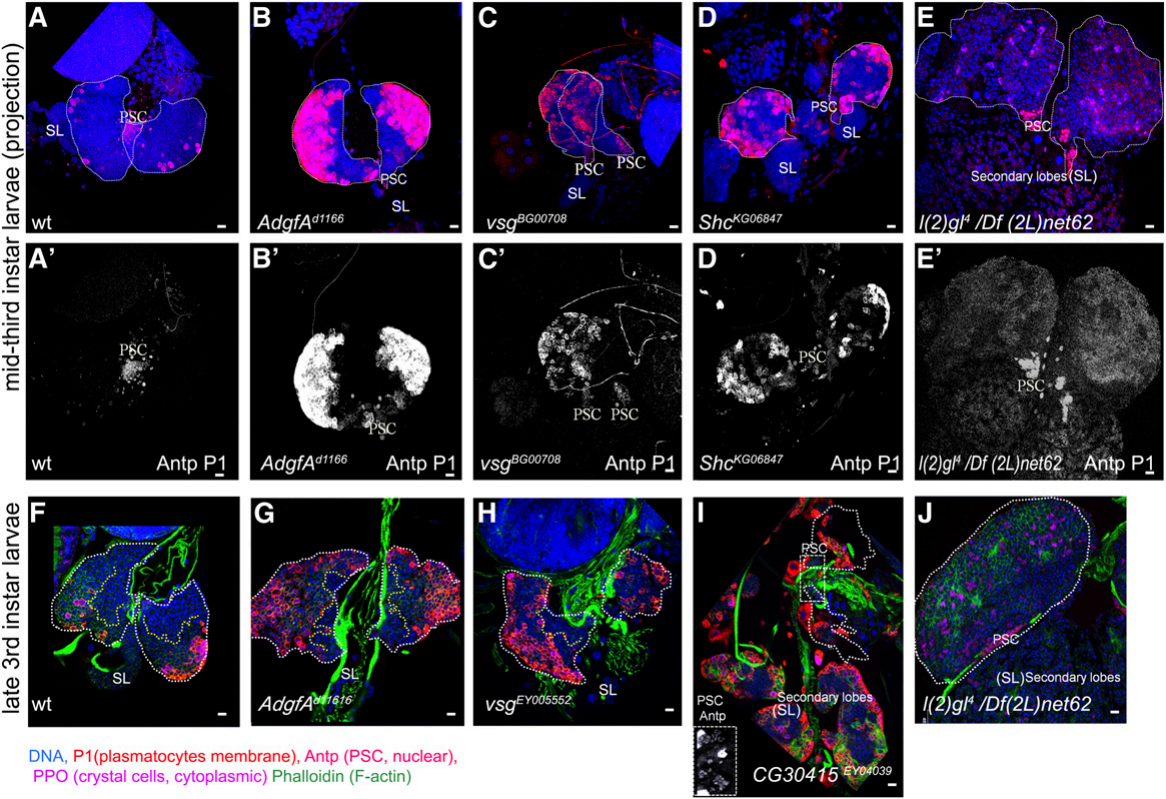
Lymph gland phenotypes of *Zfrp8^null^*/+ overgrowth suppressors. (A–E, A′–E′) Confocal projections of middle third-instar larval lymph glands. Lymph gland primary lobes are outlined by white dotted line. Normal wild-type glands have few or no plasmatocytes (P1, membrane marker, red A, A′). The number of plasmatocytes in *Agdf-A^d11616^*, *vs.g^BG00708^*, and *Shc^K66847^* homozygous mutants (B–D, B′–D′) is drastically increased, compared with that in wild-type (A, A′). PSCs are stained with anti-Antp (nuclear, red). Red channel (Antp and P1) for each projection is shown in (A′–E′). Crystal cells are stained with anti-PPO (cytoplasmic marker, pink). Primary lobes are outlined with white dotted line. Secondary lobes (SL) in (A–D) do not express differentiation markers. (F–J) Confocal cross-sections through the inner layers of late third-instar larval lymph glands. The border between MZ and CZ is shown by yellow dotted line. (F) In wild-type larvae, plasmatocyte maturation is seen in the surface layers (P1, membrane marker, red) of the CZ. *Agdf-A^d11616^* and *vsg^EY05552^* lymph glands show plasmatocyte differentiation across all CZ layers and also a significant reduction of MZ size (J, H). (I) *CG30415^EY040039^* caused an increase in plasmatocytes, disintegration of the primary lobes, and enlargement and increased differentiation of secondary lobes. The prospective positions of primary lobes (white dotted outline) were identified by the presence of the PSCs (anti-Antp, nuclear red, rectangular inset). (E, E′, J) *l(2)gl^4^/Df(2L)net62* causes lymph gland overgrowth with relatively weak staining for P1 at the surface of the lobes. Staining with phalloidin (green in F–J) shows cell shape allowing detection of lamellocytes and strongly stains heart tube. DNA is shown in blue. Scale bars are 10 μm.

By the middle of third instar, MZ cells become quiescent, while the cells in the CZ keep proliferating and the CZ expands till pupariation (Jung *et al.* 2005; Krzemien *et al.* 2010; Minakhina *et al.* 2011). The CZ contains hemocytes at different stages of differentiation. The majority of these cells are called intermediate progenitor cells (Krzemien *et al.* 2010) or CZ prohemocytes (Minakhina *et al.* 2011) (Figure 1, yellow). These cells express the intermediate marker, peroxidasin (Pxn), do not express terminal differentiation markers, and are able to proliferate (Jung *et al.* 2005; Krzemien *et al.* 2010; Nelson *et al.* 1994). During the late third instar and metamorphosis, hemocytes mature into plasmatocytes, crystal cells, and lamellocytes, and they are released into circulation (Grigorian *et al.* 2011) (Figure 1, C–E). In normal, unchallenged larvae, the maturation of CZ prohemocytes into plasmatocytes, the predominant blood cell type, starts at middle-to-late third instar at the surface of the CZ (Figures 1C and 2, A, A′, and F) and culminates at pupariation (Grigorian *et al.* 2011; Jung *et al.* 2005; Kurucz *et al.* 2007a,b; Minakhina *et al.* 2011). Crystal cells are seen in a scattered pattern throughout the CZ (Figures 1, 2, and 6). The third type of *Drosophila* blood cells, the large and flat lamellocyte, is rare in normal lymph glands. Upon immune challenge, the numbers of mature plasmatocytes as well as lamellocytes increase substantially, and the lymph gland often disintegrates before pupariation (Figure 1, D and E) (Markus *et al.* 2009; Rizki and Rizki 1992; Sorrentino *et al.* 2002).

## Identification of genes controlling normal hematopoiesis

Recently we reported a novel conserved factor, Zfrp8, that functions in *Drosophila* hematopoiesis. *Zfrp8* mutants show a loss of the MZ-CZ organization and vast overproliferation of CZ prohemocytes, leading to an increase in all three types of hemocytes, particularly lamellocytes (Minakhina *et al.* 2007; Minakhina and Steward 2010). In *Zfrp8* heterozygous animals, the size of the lymph gland is 2–2.5 times enlarged. Using clonal analysis, we showed that Z*frp8* is required in early lymph gland development for the viability or division of HSCs and that the lack of *Zfrp8* does not cause cell-autonomous overproliferation (Minakhina and Steward 2010). Therefore, loss of one or both copies of *Zfrp8* changes the homeostasis within the lymph gland and causes overproliferation of hemocytes at intermediate stages of differentiation in noncell autonomous manner.

Previously we showed that the *Zfrp8^null^*/+ lymph gland phenotype can be modified by dose reduction of genes that control cell proliferation and by genes functioning in hemocyte development (Minakhina *et al.* 2007, 2011). We identified two enhancers, Dgrip91, which involved in anchoring gamma-Tubulin to the centrosome, and Cdc27, which is a component of the anaphase-promoting complex (APC). The GATA factor gene *pannier (pnr*) was identified as a suppressor of *Zfrp8^null^*/+ and *Zfrp8^null^/Zfrp8^null^* lymph gland overgrowth (Minakhina *et al.* 2007). Further studies have shown that *pnr* is required for plasmatocyte differentiation and that the balance between the two Pnr isoforms, Pnr α and β, controls hemocyte proliferation (Minakhina *et al.* 2011). This finding suggested that *Zfrp8^null^*/+ lymph gland overgrowth may be manipulated by changes in cell proliferation as well as differentiation.

We performed a genetic deficiency screen designed to identify modifiers of the dominant *Zfrp8^null^*/+ lymph gland phenotype. We found 21 deficiencies that altered the phenotype. Further analysis of 9 of these deficiencies identified 11 suppressors and four enhancers of *Zfrp8^null^*/+ lymph gland overgrowth. Two of the enhancers, *mutagen-sensitive* 304 (*mus304)* and *no poles (nopo*) are reported regulators of the cell cycle. The genes suppressing *Zfrp8* overgrowth mostly controlled plasmatocyte differentiation and changed the homeostasis between prohemocytes and mature blood cells.

The large number of deficiencies and new genes identified suggest that our screen is far from saturated and that only a small portion of the genes functioning in *Drosophila* hematopoiesis are known so far. We describe the hematopoietic phenotypes of 10 genes. Of these, 9 genes were not previously reported to function in *Drosophila* hematopoiesis, and 4 of them have mammalian orthologs with a role in blood development and leukemogenesis. Thus, hematopoiesis in flies is more complex than previously demonstrated, and in its complexity it is even more similar to blood formation in vertebrates.

## MATERIALS AND METHODS

### *Drosophila* strains, collection of larvae, and *Zfrp8* genetic interactions

*Zfrp8^Df(2R)SM206^* was used as the *Zfrp8^null^* allele (Minakhina *et al.* 2007; Minakhina and Steward 2010). Fly stocks bearing the mutations *nopo^Z1447^* and *nopo^null^* (*nopo^Exc142^*) were kindly provided by Dr. L. A. Lee (Vanderbilt University, Nashville, TN); and *pxn-GAL4* and *2x-UASGFP* by Dr. U. Banerjee (University of California, Los Angeles, CA). Collection of deficiencies uncovering the second and third chromosomes of the genome, drivers, balancer stocks, smaller deficiencies, mutant alleles, and *P*-element insertions were obtained from the Bloomington Drosophila Stock Center (Bloomington, IN). *UAS-dsRNA* (RNAi) transgenic lines were obtained from the Vienna Drosophila Resource Center (VDRC) and the Bloomington Drosophila Stock Center (TRiP lines) (Dietzl *et al.* 2007; Ni *et al.* 2011). Deficiencies, mutant alleles, and RNAi lines are listed in Tables 1 and 2 and Table S1. *tub-Gal4 UAS-dcr2/TM6b* was used for strong general gene knockdown, and *dome-GAL4/FM7*, *UAS-cd8GFP* was used for MZ visualization and knockdown in the MZ. *T(2;3)B3*, *CyO;TM6B Tb^1^*, *Gla^BC^*, and GFP-marked balancer chromosomes were used to identify mutant larvae. *w*118, *driver/+*, and *UAS-RNAi* animals were used as wild-type controls.

**Table 1:** Summary of the screen for genetic modifiers of *Zfrp8/*+ lymph gland phenotype

Deficiency Modifying *Zfrp8/+* Lymph Gland Overgrowth	Deleted Segment, Cytology	Numbers of Lymph Glands with Phenotype/ Total Analyzed	Comment on Mapping	Modifying Alleles and RNAi Lines
Suppressing				
* Df(2L)BSC31*	23E5–23F5	7/13	Smaller deficiencies tested, no modifiers found.	n/i
* Df(2L)BSC17*	30C3–30F1	14/14	Mapped to 30C1 and 30C5. Two dominant suppressors identified.	*pelo*^1^, *hoip*^k07104^
* Df(2R)cn9*	42E–44C1	20/27	Not mapped.	n/i
* Df(2R)BSC29*	45D3–45F6	7/12	Mapped to 45F5–46A1.	*CG30338* {v 29189}
* Df(2R)en-A*	47D3–48B2	6/11	Not mapped.	n/i
* Df2R)BSC11*	50E6–51E4	10/10	Mapped to 51A5—51C1.	n/i
* Df(2R)PC4*	55A1–55F2	9/10	Mapped to 55B11–55C9.	*nopo* {v104477} *Idgf5* {v100977}
* Df(2R)BSC22*	56D7–56F12	10/19	Not mapped.	n/i
* Df(2R)vir130*	59B1–59E1	17/17	Smaller deficiencies tested, no modifiers found.	n/i
* Df(2R)or-Br6*	59D5–60B8	13/24	Weak modifier mapped to 59D9.	*CG30415^EY04039^*
* Df(2R)M60E*	60E6–60E11	20/21	Not mapped.	
* Df(3L)BSC23*	62E8–63B6	8/12	Not mapped.	
* Df(3L)AC1*	67A2–67D13	10/14	Two dominant suppressors identified.	*shc^KG06847^*, *vs.g ^EY05552^*, *MTF-1 {v107124}*
* Df(3L)Pc-2q*	78C5–79A1	9/15	Not mapped.	n/i
* Df(3R)mbc-30*	95A5–95C11	9/10	Mapped to 95B1–95D1.	n/i
				
Modifying			Phenotype of *trans*-heterozygous lymph glands. Comment on mapping.	
* Df(2L)net-PMF*	21A1–21B8	9/12	Small CZ. Both enhancer and suppressor are found.	*l(2)gl^4^*, *smo^3^*
* Df(2R)Egfr5*	57D2–58D1	9/9	Small lymph gland with changed morphology. Not mapped.	n/i
* Df(3L)emc-E12*	61A–61D3	21/28	Lymph glands are enlarged and disintegrated. Enhancing region mapped to 61A5–61B1 and one suppressing gene mapped to 61B1.	*Pk61C^EP3091^*, *Pk61C^5^*,
* Df(3L)W10*	75A6–75C2	lethal	Smaller deficiencies do not show lethality. Enhancing region is mapped to 74D1–75B11 Enhancer and suppressors found.	*Eip75B*^A81^, *Adgf-A^d11616^*, *mus304*^D3^, n/i
* Df(3L)BSC249*	79B2–79D1	11/11	Disintegrated primary lobes. Not mapped.	n/i
* Df(3R)ME15*	81F3–82F7	14/14	Disintegrated primary lobes. Not mapped.	n/i
				
*Zfrp8*-independent phenotypes			Lymph gland phenotype of deficiency/+	Comment
* Df(2L)ast2*	21D1–22B3	15/15	Lymph gland disintegration mapped to 21D4-21E2.	*Ush*, *lwr* are known to cause hematopoietic phenotype
* Df(2L)c144*	22F4–23C3	15/17	Increased size and differentiation.	
* Df(2L)spd^j2^*	27B2–27F2	10/10	Decreased size and small CZ.	
* Df(2R)cn9*	42E–44C1	27/27	Decreased size, increased differentiation.	
* Df(2L)TW161*	38A6–40B1	8/11	Decreased size, increased differentiation.	
* Df(3L)XG5*	71C2–72C1	19/19	Enlarged cortex.	
* Df(3R)BSC38*	85F1–86C8	6/10	Enlarged.	
* Df(3R)T-32*	86D9–87C4	13/17	Enlarged disintegrated.	
* Df(3R)ea*	88E7–89A1	18/18	Enlarged disintegrated.	
* Df(3R)crb87-5*	95F8–96A20	20/20	Primary lobe enlargement.	

Columns 1 and 2 show the deficiencies that modify the *Zfrp8^null^*/+ phenotype in more than 50% of the lymph glands and their cytological location. Column 3 shows the number of lymph glands with modified phenotypes relative to the total number analyzed. Column 4 shows the smallest cytological interval to which the modifying effect was mapped. Column 5 lists the genes that were found to have modifying effect. n/i, modifying genes were not identified.

**Table 2:** Alleles and RNAi lines tested for modification of *Zfrp8^null^*/+ lymph gland phenotype

Genomic Region	Number of Annotated Genes	Tested Alleles	Tested RNAi Lines
21A1–21B8	∼50	*kis^1^* *spen^3^* *smo^3^* *l(2)gl^4^* *Nhe^DP01088^*	*mbm* {v18699} *CG17075* {v103741} *CG33635* {v48812} *CG11604* {v18699}
30C1	5		*GlcAT-S* {v42779} *Trx2* {v104629},
30C5	6	*pelo^1^* *hoip^k07104^* *Pka-C1^DN^*	
45F5–46A1	16	*updo^KG01041^* *mmp2^k00604^* *Map60^KG00506^*	*Not1* {JF01135} *RpL31*{v104467} *CG30338* {v29189} *CG30340* {v100088}
51A5–51C1	∼30	*Tra2*^B^ *ttv^00681b^* *ttv^G00158^* *Sec61β^07214^* *Uhg5^EY04055^*	*Sec61β* {v107784} *Su(var)2-HP2* {JF01994} *M-spondin* {v107608} *CG12858* {v1688}
55B11–55C9	∼60	*Ote^B279^* *lolal^k02512^* *sbb^BG01610^* *Dp1^BG02288^* *imd^BG02288^* *fj^dl^*	*nopo* {v104477} *lolal* {JF01419} *pen-2* {JF02608} *sbb* {JF02375} *Idgf5* {v100977}
59D9	9	*CG13551*^e03979^ *CG30415*^EY04039^	
61A5–61D3	∼130	*Pk61C^EP3091^* *CG13887^EY2104^* *thoc7^G4736^* *CG16940^G4736^*	*NitFhit* {v27830} *pyx* {JF01242}, *CG13887* {v106452} *mri* {JF01921}
67A2–67D13	∼150	*Shc^KG06847^* *Uch-L3j^2B8^* *phol^81A^* *vsg^EY05552^* *vsg^EY05552^* *fry^7^* *nbs^1^* *RpS17^4^* *NF-YA^KG06786^* *CG13887^EY21004^*	*MTF-1* {v107124} *CG3911*, {21738} *NF-YA* {JF02013} *LanB2* {v104013} *CG33696* {v101076} *CG16719* {102653} *CG18177* {101408}
74D1–75B11	∼90	*Eip75B^Δ51^* *Adgf-A^d11616^* *mus304^D3^* *mus304^D1^* *NUCB1^c01508^* *CG5589^f06152^*	

Seventy genes from 10 genomic regions were tested for their ability to modify the Zfrp8/+ lymph gland phenotype. Column 2 shows the approximate number of annotated genes in each region. Columns 3 and 4 show the alleles and RNAi lines tested for modifying effect. The ID of each RNAi line is shown in braces.

Larvae were grown on standard cornmeal/molasses food at 25° until they reached the third-instar larval stage. Late third-instar larvae were collected 6–12 hr before pupariation. Middle third-instar larvae were collected 24–36 hours before pupariation. Mutant larvae that failed to pupate were timed according to the development of their heterozygous siblings. Gut color, mouth hook and spiracle morphology, and the size and morphology of the brain and discs served as additional controls for developmental staging.

The genetic screen was based on the mild haplo-insufficient phenotype of *Zfrp8^null^/+* larvae. The lymph gland of *Zfrp8^null^/+* larvae is on average twice (2.1; SD = 0.2) as large as that of wild type. Second and third chromosome deficiencies were crossed to *T(2;3)B3*, *CyO*; *TM6B*, *Tb^1^* balancers and then crossed with *Zfrp8^null^ /T(2;3)B3*, *CyO*. From each cross, 10–30 *trans*-heterozygous, non-*Tb* larvae at the late third-instar stage were dissected, the area of 2D images of sample lymph glands were measured using Adobe PhotoShop and normalized to the size of the wild-type gland as previously described (Minakhina *et al.* 2007).

### Immunochemistry and imaging

Larval lymph glands were dissected, fixed, immunostained, and analyzed as described previously (Jung *et al.* 2005; Minakhina and Steward 2010; Minakhina *et al.* 2011). Antibodies specific for plasmatocytes (P1, 1:400 dilution; from Dr. I. Ando, Biological Research Center, Szeged, Hungary); rabbit anti-PPO2 antibody (1:2000; from Dr. G. Christophides, Imperial College, London); rabbit anti-Pxn antibody (1:700; from Drs. John Fessler and Sergey Sinenko, UCLA); and anti-Antp antibody (1:20; from Glicksman and D. Brower, Developmental Studies Hybridoma Bank, University of Iowa, Iowa City, IA) were used as plasmatocyte, crystal cells, CZ prohemocyte, and PSC markers, respectively (Christophides *et al.* 2002; Kurucz *et al.* 2007a, b; Nelson *et al.* 1994). Alexa Fluor 488 phalloidin (Invitrogen, Carlsbad, CA) was used to visualize cell shape and detect lamellocytes. Secondary goat anti-mouse Cy3 and goat anti-rabbit Cy5 (Jackson ImmunoResearch Laboratories, West Grove, PA) were used at 1:500. DNA was stained using Hoechst 33258 (Invitrogen, Carlsbad, CA). Samples were mounted in Vectashield (Vector Laboratories, Burlingame, CA) and examined with a Zeiss Axioplan-2 microscope. Images were captured using a Leica DM IRBE laser scanning confocal microscope (objectives 40× and 63× oil), analyzed with Leica Microsystems software, and further processed using Adobe PhotoShop.

## RESULTS

To find genetic interactors of *Zfrp8* and identify novel genes functioning in hematopoiesis, we performed a dominant modifier screen. We took advantage of the dominant phenotype of *Zfrp8^null^ /+* larvae, whose lymph glands are 2.1 (SD = 0.2) times larger than wild type, and a series of chromosomal deficiency lines (Bloomington Drosophila Stock Center) that uncover more than half of the genome. Two-hundred eighty-eight second and third chromosomal deficiencies (Table 1 and Table S1) were crossed with *Zfrp8^null^/T(2:3)Cy*, *Tb* to obtain *Df +/+ Zfrp8^null^* or *Zfrp8^null^/+*, *Df/+* larvae. Then 10–30 *trans*-heterozygous late third-instar larvae from each cross were dissected and the size of the lymph glands assessed [see *Materials and Methods* and Minakhina *et al.* (2007)]. When more than half of the lymph glands were in the range of 0.8–1.2 times the size of the wild-type gland (average size of all glands 1–1.3; SD < 0.3), the deficiency was classified as a strong suppressor (Table 1). Weak modifiers that altered the average size of the *Zfrp8/+* lymph gland in fewer than half of the animals and showed variations in shape or size are listed in Table S1. The majority of deficiencies tested (190) *in trans* to *Zfrp8^null^* show no difference from *Zfrp8^null^* /+ lymph gland phenotype (Table S1).

We identified 18 deficiencies that strongly suppress the *Zfrp8^null^ /+* lymph gland overgrowth. The morphology of the lymph gland of these heterozygous deficiencies was also analyzed in a *Zfrp8^+^* background. Three deletions produced smaller lymph glands than normal in heterozygous animals (“*Zfrp8*-independent phenotypes”; Table 1). The other 15 deletions caused no or a moderate enlargement (from 1.2 to 1.7 the wild-type size) of the lymph gland (see “Suppressing” in Table 1, column 1).

We also identified 13 deficiencies that changed the shape of *Zfrp8^null^*/+ lymph glands (more than half of the glands show severe reproducible changes). These changes included loss of the petal-shaped CZ (Figure 1F), altered CZ shapes (Figure 1G), lymph gland disintegration (Figure 1, D and E), and primary or secondary lobe enlargement. When crossed to wild type, 7 of them showed the same morphological changes as observed *in trans* to *Zfrp8* and were placed into the “*Zfrp8*-independent” group (Table 1). The other 6 deficiencies showed a phenotype only *in trans* to *Zfrp8^null^* and showed normal morphology in a wild-type background. They formed a group called “modifying deficiencies” (Table 1). This group also included *Df(3L)W10*, which caused lethality of *trans*-heterozygotes (*Zfrp8^null^*/+, *Df(3L)W10*/+).

Smaller and partially overlapping deficiencies in 12 of the 32 chromosomal regions were checked for their ability to modify the *Zfrp8^null^/+* phenotype. Smaller deletions in regions 23E–23F and 59B–59E showed no phenotypic change *in trans* to *Zfrp8^null^*. From the other 10 chromosomal regions, each uncovering from 5 to 150 genes, we chose 41 genes that had available mutant alleles (Table 2). Priority was given to genes that did not have an established function in *Drosophila* hematopoiesis. Lymph glands of *trans*-heterozygous *Zfrp8^null^* larvae were scored as above. Smaller deficiencies uncovering the segment deleted in *Df(3L)W10* did not allow us to identify the region responsible for the lethality. However, one of the deletions, *Df(3L)ED4710*, showed enlargement of the *Zfrp8^null^*/+ lymph gland: 9 of 16 lymph glands were 3.5 to 4 times larger than wild type.

### Dominant suppressors of *Zfrp8* regulate hemocyte differentiation

**Figure 3:**
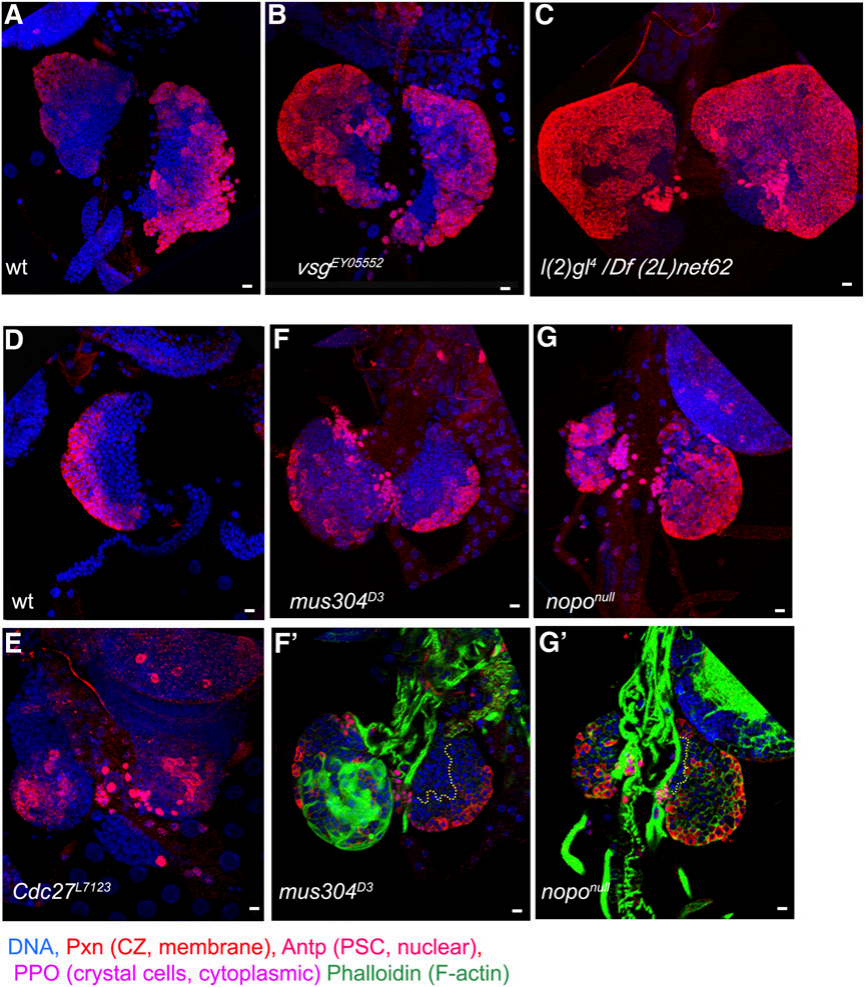
Changes in MZ-CZ organization in mutant lymph glands. Confocal projections of late third-instar lymph glands of wild type (A), *vsg^EY05552^* (B) homozygous mutants, and *l(2)gl^4^ Df(2L)net62* (C). The relative size of the CZ (anti-Pxn, membrane-cytoplasmic, red) in both mutants is increased, whereas the MZ (Pxn-negative) size is decreased. Severe reduction of the MZ is also seen in early middle third-instar *nopo^null^* lymph glands (G, G′) compared with wild-type lymph glands at the same stage (D). In *Cdc27^L7123^* and *mus304^D3^* mutants (E, F) the relative sizes of MZ and CZ are not significantly changed. Staining with phalloidin (green) shows the burst of lamellocytes in cross-sections through the inner lymph gland layers (F′, G′) in one lobe of *mus304^D3^*. Yellow dotted line separates MZ and CZ in (F′, G′). PSCs are stained with anti-Antp (nuclear red). DNA is shown in blue. Scale bars are 10 μm.

**Figure 4:**
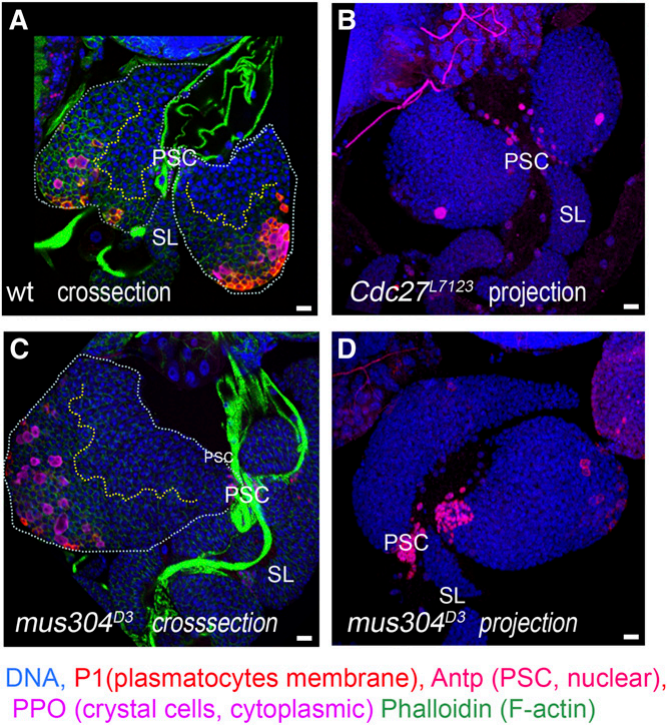
Lymph gland differentiation in *mus304* and *cdc27* mutants Confocal cross-sections (A, C) and projections (B, D) of wild type (A), *Cdc27^L7123^* (B), and *mus304^D3^* (C, D) middle-late third-instar larval lymph glands. Mutant lymph glands show some delay in plasmatocyte differentiation (P1, membrane marker, red). *mus304^D3^* lymph glands show variations in size and differentiation (C, D). PSCs are stained with anti-Antp (nuclear red). DNA is shown in blue. (A, C) The primary lobes are outlined by white dotted line, and the border between MZ and CZ is shown by yellow dotted line; staining with phalloidin (green in A, C) allows detection of lamellocytes. Scale bars are 10 μm.

The screen for dominant modifiers identified both known and novel genes involved in *Drosophila* hematopoiesis and immunity. For example, *smoothened* (*smo*) and *adenosine deaminase growth factor* (*Adgf-A*) were known to function in the development of hemocytes, and *signal peptide protease* (*spp*) is known to regulate *Drosophila* immunity (Dolezal *et al.* 2005; Junell *et al.* 2007; Tokusumi *et al.* 2010). But the nine genes [*pelota (pelo*), *hoi-polloi (hoip*), *mutagen-sensitive 304* (*mus304)*, *SHC-adaptor protein (Shc*), *visgun (vsg*), *phosphoinositide-dependent protein kinase 1 (Pdk1)/Pk61C*, *ecdysone-induced protein 75B* (*Eip75B*), *CG30415*, and *lethal (2) giant larvae* (*l(2)gl*)] did not have an established function in hematopoiesis. To determine a possible hematopoietic function of these genes, we analyzed the lymph gland phenotypes of homozygous mutant larvae at early middle and late third-instar stages using specific markers for terminal hemocyte maturation. We used P1 antibody staining the plasmatocyte-specific protein Nimrod C1 (Kurucz *et al.* 2007a), anti-Pro-phenoloxidase for crystal cells (Christophides *et al.* 2002; De Gregorio *et al.* 2002), phalloidin to reveal the shape of lamellocytes, and anti-Pxn to define the CZ (Figures 2–4) (Jung *et al.* 2005; Nelson *et al.* 1994). Homozygotes of the suppressing genes *hoip* (*hoip^k07104^*) and *Pdk1*/*Pk61C (Pk61C^EP3091^)* showed early lethality and were excluded from this analysis. Lymph glands of middle and late third-instar *Adgf-A*, *vsg*, *shc*, and *CG30415* homozygous larvae were compared with wild-type lymph glands at similar stages of development (Figure 2).

During normal development, the crystal cells can be detected in the CZ throughout the third-instar larval stages. Lamellocytes are only occasionally seen in late third-instar larval lymph glands. The Nimrod C1 (P1)-positive plasmatocytes are not seen in the lymph glands until the middle of third instar. During late third-instar larval development, plasmatocyte differentiation starts on the surface of the CZ, whereas the hemocytes in the inner layers of the CZ differentiate later during metamorphosis (Figures 1C and 2F) (Grigorian *et al.* 2011; Minakhina *et al.* 2011).

*Adgf-A* controls deamination of adenosine and deoxyadenosine. Complete loss of *Adgf-A* causes a dramatic increase in levels of adenosine and deoxyadenosine in larval hemolymph. It also leads to increased numbers of circulating hemocytes, disintegration of the lymph gland primary lobe, a large number of cells with characteristics of plasmatocytes and macrophages, and larval death (Dolezal *et al.* 2005). We found that a hypomorphic allele, *Adgf-A^d11616^*, caused by a *P*-element insertion into the first intron, did not affect viability but caused a strong hematopoietic phenotype (Figure 2, B, B′, and G). Plasmatocyte differentiation starts prematurely in early third-instar larvae, and by the late third-instar stage, the majority of cortical prohemocytes, including the hemocytes in the inner layers of the gland, were positive for the plasmatocyte marker. The size of the medulla was significantly reduced compared with wild type (Figure 2G).

*Vsg* is an ortholog of mammalian sialomucin endolyn (CD164), an adhesion receptor protein that regulates the adhesion of CD34^+^ stem or precursor cells to bone marrow stroma and the recruitment of CD34^+^ cells into mitosis (Chan *et al.* 2001; Forde *et al.* 2007). In *Drosophila*, all existing loss of function alleles are homozygous viable. Although ablation of Vsg inhibits proliferation in S2 cells and loss-of-function mutations cause some reduction in embryonic viability, it seems otherwise not to be required for animal development and fecundity (Zhou *et al.* 2006). The function of *vsg* in *Drosophila* hematopoiesis or immune response has not been previously studied. We tested the phenotypes of two *P*-element alleles, hypomorphic *vsg^EY0552^* and complete loss-of-function *vsg^BG00708^*. Both alleles showed increased plasmatocyte differentiation, detectible in early-to-middle third-instar larvae, and by late third instar, the majority of cortical cells expressed the plasmatocyte marker (P1, membrane red in Figure 2, C, C′, and H). In the mutants, the size of the MZ was decreased, whereas the CZ became relatively enlarged (Figures 2H and 3B). These results suggest that, similar to mammalian Endolin, *Drosophila* Vsg has a blood-specific function: it controls homeostasis between MZ and CZ and inhibits premature plasmatocyte maturation.

Shc is a conserved adaptor protein implicated in several signaling pathways (RAS-MAPK, EGFR, PDGFR, TrkA) (Bonati *et al.* 2000; Hallberg *et al.* 1998; Lunghi *et al.* 2001; Luschnig *et al.* 2000). In humans, Shc is highly enriched in primary acute myeloid leukemia blasts (Bonati *et al.* 2000; Lunghi *et al.* 2001). In *Drosophila*, mutations in *shc* are semi-lethal and sterile, but the function of the gene in hematopoiesis has not been studied (Luschnig *et al.* 2000). We found that a viable *P*-element insertion in the 5′-UTR of *shc* led to premature plasmatocyte differentiation seen in middle third-instar lymph glands (Figure 2, D and D′).

*CG30415* encodes the least-conserved protein that we obtained in our screen, and its function is not known. A *P*-element insertion into the 3′-UTR of *CG30415* (*CG30415^EY04039^*) causes a strong increase in plasmatocyte differentiation, leading to early disintegration of the primary lymph gland lobes and overgrowth and differentiation of the secondary lobes (Figure 2I).

*l(2)gl* encodes a component of the *Par (partitioning defective)* complex and is required for asymmetric cell division and establishment of epithelial cell polarity (Ohshiro *et al.* 2000; Peng *et al.* 2000). At the end of the third instar, *l(2)gl4/Df(2L)net62* larvae develop tumors in the brain and imaginal discs (Mechler *et al.* 1985). These mutant larvae do not enter pupation. We noticed that lymph glands also undergo strong overgrowth, up to 20–30 times depending on the age of the larva. To standardize our investigations, we analyzed the lymph gland phenotypes of homozygous mutants, heterozygous siblings, and wild-type larvae of the same age (see *Materials and Methods*). In the mutants, the lymph gland enlargement was already seen after the second molt in early third-instar larvae when the tumors in other tissues were not apparent. The CZ of the gland was significantly expanded, but it showed more variation in size and differentiation than other mutants (Figures 2, E, E′, and J, and 3C). The lymph glands in heterozygous *l(2)gl^4^/+* larvae were also slightly enlarged (not shown).

### Enhancers of the Zfrp8*/+* phenotype

In our screen, we identified two enhancers of *Zfrp8^null^*/+ lymph gland overgrowth: *smo* and *mus304* *in trans* to *Zfrp8^null^* caused substantial increase of the lymph gland size (more than four times wild-type size). A similar phenotype was previously observed in *Zfrp8^null^/+*; *Cdc27 ^L7123^*/+ animals (Minakhina *et al.* 2007).

Smo is a 7-transmembrane G-like protein-coupled receptor, an essential component of the Hh pathway known to control prohemocyte homeostasis (Mandal *et al.* 2007; Tokusumi *et al.* 2010). The homozygous *smo^3^* phenotype could not been studied because of early larval lethality. Heterozygous *smo^3^*/*+* larvae did not show lymph gland enlargement (data not shown). The study of this gene will have to be done in combination with other pathway players and will provide better understanding of the role of Hh signaling in hematopoiesis.

Mus304 is involved in the mitotic cell cycle, DNA repair, and the DNA damage checkpoint (Bi *et al.* 2005; Boyd *et al.* 1981; Brodsky *et al.* 2000). Both alleles tested, *mus304^D1^* and *mus304^D3^* *in trans* to *Zfrp8^null^*, lead to lymph gland enlargement of up to four times that of wild type. *mus304^D3^* is a homozygous viable allele that shows sensitivity to mutagens, defects in DNA repair, and telomere maintenance (Bi *et al.* 2005; Boyd *et al.* 1981; Brodsky *et al.* 2000). About half (9 of 14 tested) of the *mus304^D3^* animals show lymph gland enlargement (Figure 4, B and C), which was often accompanied (6 of 9) by lamellocyte over-differentiation in one or both primary lobes (Figure 3, F and F′). No consistent changes in medulla-cortex organization, plasmatocyte, or crystal cell differentiation were observed (Figure 4, C and D). The overgrowth and occasional lamellocyte differentiation may reflect an immune reaction to increased numbers of cells dying from unrepaired spontaneous DNA damage. In addition, some increase in cell proliferation may result from the lack of a mitotic checkpoint.

Cdc27, a previously identified enhancer of *Zfrp8^null^/+* lymph gland overgrowth, also encodes a cell-cycle protein and regulates the spindle checkpoint (Deak *et al.* 2003; Minakhina *et al.* 2007). Mutation in *Cdc27* (*Cdc27^L71123^*) caused a modest reduction in differentiation and no visible change in growth (Figures 3E and 4B).

### Identification of genes functioning in lymph gland development using conditional knockdown

**Figure 5:**
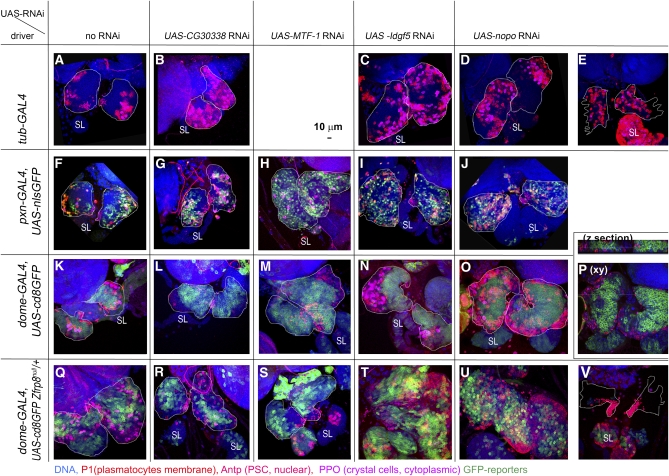
Knockdown phenotypes of *Zfrp8* modifiers. Confocal projections of lymph glands obtained from the crosses of the three drivers [*tub-GAL4* (A–E); *pxn-GAL4*,2X*UAS-nlsGFP* (CZ cells, nuclear green, F–J), and *dome-GAL4*, *UAS-cd8GFP* (MZ cells, membrane green, K–P)] to UAS-RNAi lines of *CG30338* (B, G, L, R), *MTF-1* (H, M, S), *Idlg5* (C, I, N, T), and *nopo* (D, E, J, O, P, U, V). Drivers crossed with wild type (A, F, K) show normal MZ-CZ organization and hemocyte differentiation. (Q) *dome-GAL4*, *UAS-cd8GFP* combined with heterozygous *Zfrp8^null^* shows lymph gland enlargement, similar to that of *Zfrp8^null^*/+, and increase in dome-positive cells intermingled with CZ prohemocytes. The lymph gland overgrowth is suppressed when *CG30338* (R) or *MTF-1* (S) are knocked down in MZ, whereas overgrowth is enhanced when *Idgf5* (T) or *nopo* (V) are depleted. *nopo* knockdown *in trans* to *Zfrp8^null^/+* also causes disintegration of primary lobes in late third-instar larvae (V). Primary lobes (outlined by white dotted line) are identified by their anterior position and presence of PSC (anti-Antp, nuclear red). Secondary lobes (SL) show excessive growth and differentiation in most *nopo* knockdown lymph glands (E, J, U, V). Plasmatocytes are stained with P1 antibody (membrane, red) and crystal cells with anti-PPO2 (cytoplasmic, pink). DNA is shown in blue. Scale bar is 10 μm.

No mutations were available for a large number of genes uncovered by the *Zfrp8*-interacting deficiencies. To test their function in hematopoiesis, we obtained *UAS-dsRNA* (RNAi) lines (VDRC or TRiP; Table 2) for 30 genes from seven interacting regions. Using the hemocyte-specific driver *pxn-GAL4*, we knocked down each gene in the CZ of the lymph gland and assessed the resulting phenotypes. For about half of the lines, we also utilized a general driver, *tubulin (tub)-Gal4*, and *dome-GAL4*, a driver expressed in embryos, some larval tissues, and highly expressed in most MZ cells. Few *dome*-positive cells are also found in the CZ transition zone (Sinenko *et al.* 2009). This zone is notably enlarged in *Zfrp8^null^*/+ lymph glands, and mitotically active CZ prohemocytes are intermingled with *dome*-positive cells [see Figure 5Q and Minakhina *et al.* (2007)].

The majority of the RNAi lines used in this study caused no or a variable lymph gland phenotype when expressed under the control of *pxn-Gal4*. For several genes, the strong effect of the knockdown in the CZ correlated with the general function of the gene in cell metabolism or cell survival. For instance, knockdown of *RpL31* by *pxn-GAL4* resulted in disintegration of the CZ and hemocyte death. General knockdown of *RpL31* by *tub-GAL4* caused growth arrest and early larval lethality. Similarly, *CG3911* and *Not1 RNAi* expression both caused early lethality when generally expressed (*tub-GAL4*) and showed vast reduction in numbers of CZ cells when driven by *pxn-GAL4* (not shown).

We selected four lines that triggered reproducible changes in lymph gland development and also modified the *Zfrp8^null^*/+ phenotype (Figure 5) for further study. *CG30338* and *metal response element-binding transcription factor-1 (MTF-1*) suppressed *Zfrp8^null^*/+ overgrowth (Figure 5, Q–S), whereas knockdown of *imaginal disc growth factor 5* (*Idgf5*) and *no-poles* (*nopo*) enhanced the overgrowth and led to premature primary lobe disintegration in late third-instar larvae (Figure 5, T–V). Individual knockdown of each gene caused distinct abnormalities in lymph gland development.

*CG30338* encodes an uncharacterized conserved protein, a putative ubiquitin-conjugating enzyme with DUF1115 and RWD domains (Flybase annotation; Tweedie *et al.* 2009). Animals with general depletion of *CG30338* (*tub-GAL4*) survived until adulthood. The larval lymph glands were smaller and contained increased numbers of plasmatocytes and crystal cells (Figure 5B). When *CG30338* was knocked down in CZ by *pxn-GAL4* or in MZ by *dome-GAL4*, no increase in differentiation was observed. Interestingly, half of the *dome-GAL4*, *UAS-CG30338 RNAi* animals showed fewer differentiated cells within the lymph gland CZ than controls (Figure 5, F, G, K, and L). When *CG30338* was knocked down by *dome-GAL4* in *Zfrp8^null^* /+ larvae, the lymph glands appeared smaller than in *Zfrp8^null^* /+ animals and showed similar levels of differentiation as the driver control (*dome-GAL4*, *UAS-cd8GFP*; Figure 5, K, Q, and R). Thus, *CG30338* appears to control hemocyte maturation.

*MTF-1* encodes a zinc finger protein that is conserved from insects to mammals. In response to heavy metals, it activates a set of metallothionine genes (Egli *et al.* 2003; Zhang *et al.* 2001). Knockdown of the gene by *tub-GAL4* affected larval growth and led to lethality during the third instar. The lymph glands of these ailing larvae were excluded from analysis. Gene knockdown by *dome-Gal4* or *pxn-GAL4* did not alter larval growth and viability. The lymph glands of these larvae showed an increase in size (from 1.2 to 1.7 of wild-type size) and relatively low levels of differentiation (Figure 5, H, M, and S). In all 12 *pxn-GAL4*, *UAS-MTF-1-RNAi* lymph glands, we observed very few differentiated plasmatocytes, suggesting that MTF-1 may specifically regulate plasmatocyte differentiation during normal hematopoiesis.

Idgf5 has chitinase and growth factor activity and is found in the larval hemolymph (Karlsson *et al.* 2004; Kawamura *et al.* 1999), but its role in hematopoiesis, wound healing, and immune response is unclear. When *Idgf5* RNAi was driven by *tub-GAL4* or *dome-GAL4*, the lymph gland was ∼2 times enlarged and the number of crystal cells was strongly increased (Figure 5, C and N). Knockdown of the gene at later stages of hemocyte development (*pxn-GAL4*) did not increase the number of crystal cells; rather, there were fewer differentiated hemocytes than in controls, and in the same glands, the MZ was visibly smaller (Figure 5I). These results suggest that *Idgf5* may function early in nondifferentiated hemocytes to regulate their fate. The expression of *Idgf5* RNAi under the control of *dome-Gal4* resulted in strong enhancement of the *Zfrp8^null^* /+ phenotype with increase in the size of the MZ and transition zone (green in Figure 5T). Whether the change of hemocyte fate can cause an overgrowth in sensitized *Zfrp8^null^*/+ lymph glands remains unclear.

*Nopo* is a maternal effect cell-cycle regulator and encodes an E3 ubiquitin ligase (Merkle *et al.* 2009). Knockdown of the gene by three different drivers leads to three different lymph gland phenotypes. General knockdown with tub-GAL4 caused increase in differentiation and premature disintegration of the primary lobes (Figure 5, D and E). Similar to what is observed with *Idgf5* RNAi, expression of *nopo* RNAi in CZ (*pxn-GAL4*) caused a decrease of the MZ in late third-instar lymph glands without a severe increase in differentiation. When the gene was downregulated in the MZ (*dome-GAL4*), the MZ was not diminished but the medulla-cortex spatial organization was altered. The gland became more spherical in shape, and the cortical zone covered the surface of the entire gland instead of forming a distal petal-shaped structure (Figure 5, N–P). Knockdown of *nopo* by *dome-GAL4* in combination with loss of one copy of *Zfrp8* led to gland overgrowth, followed by disintegration of the primary lobes and premature differentiation of cells in the secondary lobe (Figure 5, U and V).

**Figure 6:**
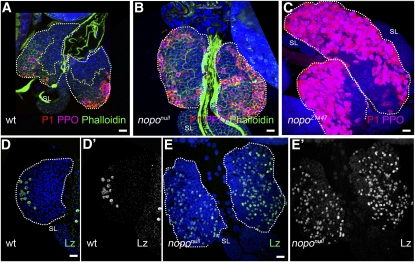
*nopo* affects lymph gland growth and hemocyte differentiation. Primary lobes are outlined with white dotted line. Crystal cell (anti-PPO2, pink) and plasmatocyte maturation (P1, membrane, red) are increased in middle third-instar (B) and late third-instar (C) *nopo^null^* lymph glands compared with that in wild type (A). Secondary lobes (SL) do not express differentiation markers. Confocal cross-sections through the inner cell layers (A, B) show drastic decrease in mutant MZ size (yellow dotted line). Staining with phalloidin (green) shows cell shape. Staining for Lozenge (Lz), an early crystal cell marker (D–E′, green/white), shows greater than 5 times increase in number of crystal cells in *nopo^null^* lymph gland lobes (E, E′) compared with that in wild type (D, D′).

General knockdown of the gene caused great reduction in larval viability, whereas existing *nopo* mutations are viable and sterile (Merkle *et al.* 2009). We analyzed the lymph gland phenotypes of two mutant alleles, *nopo^EXC142^* and *nopo ^Z1447^*. In *nopo^EXC142^*, part of the 5′-UTR and the exons encoding amino acids 1–181 are deleted, and it is therefore considered a null allele; *nopo^Z1447^* is an E11K missense mutation (Merkle *et al.* 2009). Lymph gland development in both mutant alleles was similar. The primary lobes were 3–5 times enlarged and had substantially increased numbers of plasmatocytes and crystal cells (Figure 6, B and C). The size of the MZ was greatly reduced (Figures 3, G and G′, and 6B). In second- and early third-instar larvae, the MZ was represented by a narrow strip of cells adjacent to the pericardial cells along the dorsal vessel (Figure 3G′), and during the third-instar larval stage, it was barely half the size of the wild-type MZ (Figure 6B). The mutants also showed an increase in crystal cell numbers. To test if the increase in mature crystal cells (PPO-positive) correlates with an increase in crystal-cell precursors, we stained the wild-type and mutant lymph glands for the early crystal cell marker Lz. In wild type, Lz-positive cells were relatively densely packed in the CZ of middle third-instar lymph glands. In *nopo* mutants of the same age, the CZ was greatly enlarged, and Lz-positive cells were found in a similar density all over the CZ (Figure 6, D–E′). The increase in mature crystal cells in *nopo* mutants correlated with CZ enlargement and overall early blood cell maturation within the CZ (Figure 6B). Mutant and knockdown phenotypes suggest that *nopo* controls homeostasis between the hematopoietic precursors in the MZ, and the prohemocytes and mature blood cells in the CZ.

## DISCUSSION

Analysis of Zfrp8 mutant clones in the lymph gland show that it is required in HSCs but is dispensable at later stages of hemocyte proliferation or differentiation. But Zfrp8 mutants have vast lymph gland overgrowth (Minakhina *et al.* 2007; Minakhina and Steward 2010). In the mutant lymph glands, all hemocytes rapidly proceed to the stage of differentiation corresponding to wild-type CZ prohemocytes, the most mitotically active stage during hemocyte development (Jung *et al.* 2005; Krzemien *et al.* 2010; Minakhina and Steward 2010). Therefore, it is the loss of HSCs in the mutant animals that ultimately leads to lymph gland overgrowth and severe change in hemocyte homeostasis. Interestingly, the loss of one copy of the gene also affects hemocyte development, leading to noticeable lymph gland enlargement (Minakhina *et al.* 2007). In our previous screen for candidate modifiers of *Zfrp8^null^/+* lymph gland overgrowth, we identified two cell-cycle regulators and one novel gene, *pnr*, required for plasmatocyte differentiation (Minakhina *et al.* 2007, 2011). Hence, we hypothesized that two major groups of factors contribute to the *Zfrp8* lymph gland phenotype, direct regulators of the cell cycle, and factors controlling hemocyte differentiation.

In the past, several genetic screens in other laboratories were designed to detect regulators of cellular immune response, hemocyte activation, and melanotic mass formation (Avet-Rochex *et al.* 2010; Milchanowski *et al.* 2004; Rodriguez *et al.* 1996; Zettervall *et al.* 2004). The majority of the genes identified in these screens regulate lamellocyte and crystal cell development. Our present genetic screen yielded a number of new genes involved in hemocyte differentiation, particularly of plasmatocytes. The lack of significant overlap between all screens can be explained by the difference in screen designs. It also suggests that many hematopoietic genes have not been identified so far. For instance, a recent RNAi screen by Avet-Rochex *et al.* (2010) targeting about 10% of *Drosophila* genes identified 59 hematopoietic factors, 55 of which were not previously linked to blood cell development.

In our screen, we identified 10 deficiencies that had dominant lymph gland phenotypes both in a wild-type background and *in* *trans* to *Zfrp8^null^* and 21 deficiencies that modified the *Zfrp8^null^/+* phenotype but did not show lymph gland abnormalities in a wild-type background. We tested ∼15% of the genes uncovered by 9 modifying deficiencies and identified 16 suppressors and enhancers of *Zfrp8^null^/+* lymph gland overgrowth. For 9 of these genes, we showed a new role in the regulation of blood cell development.

Our screen also suggested that the number of uncharacterized genes regulating different aspects of hematopoiesis is much larger than the number of genes identified so far. This conclusion is supported by the following observation: we characterized half of interacting deficiencies and many of them uncover two or more modifying genes. For instance, in two chromosomal regions (30C5 and 67A2–D12), we found four dominant suppressors, *pelo*, *hoip*, *shc*, *and vsg*. Other two modifying deficiencies, *Df(3L)W10* and *DF(2L)net-PMF*, contained both enhancers and suppressors (see Table 1).

Of the four enhancers identified in the screen, two, *mus304* and *nopo*, function in the DNA-damage response and cell-cycle regulation. A previously identified enhancer of *Zfrp8^null^/+* lymph gland overgrowth, *Cdc27*, also encodes a cell-cycle protein that regulates the spindle checkpoint (Deak *et al.* 2003; Minakhina *et al.* 2007). Analysis of homozygous mutant phenotypes of individual genes has shown that each of the three genes triggered different changes in lymph gland development. Mutation in *Cdc27* caused modest decrease in growth and differentiation (Figures 3E and 4B), and *mus304* prompted moderate overgrowth and high incidence of lamellocyte differentiation in one or more lobes (Figures 3, F and F′, and 4, C and D). Mutations in *nopo* and *nopo* gene knockdown had complex effects on lymph gland development, including noticeable overgrowth, MZ reduction, and increase in crystal cells (Figures 5 and 6). None of the three genes had a dominant phenotype, and all of them enhanced *Zfrp8^null^/+* lymph gland overgrowth. These genes may function together with *Zfrp8* in regulating HSC division. Alternatively, their dominant effects on cell-cycle timing may become only evident in the *Zfrp8^null^/+* background, in which the population of mitotically active CZ prohemocytes is increased.

The majority of dominant modifiers identified in the screen suppressed the *Zfrp8^null^/+* lymph gland overgrowth. We analyzed homozygous phenotypes of five of the nine suppressing genes, and all represented different molecular pathways. With the exception of *l(2)gl*, they all had a similar effect on hemocyte differentiation. Homozygous loss-of-function mutants induced early plasmatocyte differentiation and changed the balance between the CZ and MZ (Figures 2 and 3). Three of the genes, *AdgfA*, *vsg*, and *Shc*, are highly conserved across species, but only *AdgfA* was previously reported to function in *Drosophila* hematopoiesis (Dolezal *et al.* 2005). Interestingly, human homologs of both Vsg and Shc are differentially expressed in hematopoietic progenitors and leukemic cells (Chan *et al.* 2001; Forde *et al.* 2007; Fukuda *et al.* 2011; Lunghi *et al.* 2001). The mammalian Vsg ortholog, endolyn (CD164), regulates the interaction of hematopoietic stem and precursor cells with the niche and their adhesion and migration (Forde *et al.* 2007). *Drosophila* Vsg is required for homeostasis between precursor cells and differentiated hemocytes, a process that also depends on niche signaling.

Using RNAi-mediated conditional gene knockdown, we identified four new genes functioning in *Drosophila* hematopoiesis. Three of them may have similar functions in vertebrates. We found that *MTF-1* is required for plasmatocyte development in the *Drosophila* lymph gland, whereas its mouse homolog is known to play a critical role in lymphocyte development (Wang *et al.* 2004). The second gene, *nopo*, affects early embryonic development (Merkle *et al.* 2009), and in our experiments, it shows a striking lymph gland phenotype but does not affect other somatic tissues. It has been suggested that lack of Nopo may change the timing of the S-M transition and lead to acentrosomal or mis-oriented spindles (Merkle *et al.* 2009). The development of lymph glands may be particularly sensitive to these defects. However Nopo may also function in ubiquitination of non-cell-cycle–related, blood-cell–specific targets. Nopo is one of about 130 E3 ubiquitin ligases encoded in the *Drosophila* genome (Merkle *et al.* 2009), and its mammalian orthologs, such as c-Cbl, Fbw7, Triad1, and FLRF (Rnf41), are implicated in normal and malignant hematopoiesis (Jing *et al.* 2008; Kitagawa *et al.* 2009; Marteijn *et al.* 2009; Rathinam *et al.* 2008; Reavie *et al.* 2010). The third conserved gene, *CG30338*, encodes a predicted ubiquitin-conjugating enzyme, another component of ubiquitination pathway. It is the least-studied gene, and its interesting hematopoietic phenotype in flies and the conserved structure of the protein across species warrants future investigation.

Most mutations identified in this screen caused distinct abnormalities in hemocyte differentiation, but none resulted in complete loss or enlargement of the MZ, suggesting that early stages of hematopoiesis were least affected. Given that Zfrp8 is required already in the embryo in HSCs and that the overgrowth is a consequence of this early lymph gland abnormality, it is not surprising that we obtained an array of genes functioning at different points of hematopoiesis later in development, affecting a number of conserved molecular pathways. Our results show that the similarities between mechanisms of regulation of hematopoiesis in flies and mammals extend beyond transcriptional regulation, and hence, *Drosophila* serves as an excellent model for identification and study of genes functioning in growth control, cell cycle, and cell differentiation in hematopoiesis.

## Supplementary Material

Supporting Information
