# Supplementary material for: Genetic Screen for Regulators of Lymph Gland Homeostasis and Hemocyte Maturation in Drosophila
Source: G3 (Bethesda). 2012 Mar 1;2(3):393–405. doi: 10.1534/g3.111.001693 (PMC3291509; doi:10.1534/g3.111.001693)
Supplement: Supporting Information [file supp_2_3_393__index.html]

Supporting Information 

# Genetic Screen for Regulators of Lymph Gland Homeostasis and Hemocyte Maturation in *Drosophila*

## Supporting Information for Tan, Goh, and Minakhina, 2012

**Files in this Data Supplement:**

- Table S1 - Deficiencies with weak or no modifying effects on Zfrp8/+ lymph gland phenotype (PDF, 100 KB)
