# Supplementary material for: Genetic Screen for Regulators of Lymph Gland Homeostasis and Hemocyte Maturation in Drosophila
Source: G3 (Bethesda). 2012 Mar 1;2(3):393–405. doi: 10.1534/g3.111.001693 (PMC3291509; doi:10.1534/g3.111.001693)
Supplement: Supporting Information [file supp_2.3.393_TableS1.pdf]

Table S1 Deficiencies with weak or no modifying effects on Zfrp8/+ lymph gland phenotype

Deficiency	Deleted segment, cytology	Numbers of lymph glands with modified phenotype to total analyzed	Modified phenotype
Df(2L)dp-79b	22A2-3;22D5-E1	(11/20)	Enlarged, disintegrated
Df(2L)ed1	24A2;24D4	(5/10)	Enlarged,
Df(2L)BSC110	25C1;25C4	(2/10)	Disintegrated
Df(2L)cl-h3	25D2-4;26B2-5	(3/12)	Suppressed
Df(2L)E110	25F3-26A1;26D3-11	(5/14)	Enlarged
Df(2L)BSC5	26B1-2;26D1-2	(2/10)	Suppressed
Df(2L)Dwee1-W05	27C2-3;27C4-5	(2/13)	Suppressed
Df(2L)Mdh	30D-30F;31F	(7/12)	Variable
Df(2L)BSC143	31B1;31D9	(2/11)	Suppressed
Df(2L)Prl	32F1-3;33F1-2	(7/20)	Suppressed
Df(2L)BSC30	34A3;34B7-9	(4/12)	Suppressed
Df(2L)b87e25	34B12-C1;35B10-C1	(5/14)	Enlarged, disintegrated
Df(2L)BSC147	34C1;34C6	(2/11)	Suppressed
Df(2L)cact-255rv64	35F-36A;36D	(3/13)	Suppressed
Df(2L)Exel6049	40A5;40D3	(2/13)	Suppressed
Df(2L)C'	h35-40A1	(3/12)	Disintegrated
Df(2R)M41A4	41A;41A	(4/10)	Variable
Df(2R)nap9	42A1-2;42E6-F1	(4/15)	Suppressed
Df(2R)ST1	42B3-5;43E15-18	(3/10)	Suppressed
Df(2R)H3E1	44D1-4;44F12	(6/12)	Enlarged, disintegrated
Df(2R)BSC132	45F6;46B4	(2/10)	Suppressed
Df(2R)en30	48A3-4;48C6-8	(8/14)	Variable, altered CZ
Df(2R)BSC3	48E12-F4;49A11-B6	(2/10)	Suppressed
Df(2R)vg-C	49A4-13;49E7-F1	(2/11)	Suppressed

Df(2R)BSC44	54B1-2;54B7-10	(3/10)	Suppressed
Df(2R)k10408	54B16;54B16	(21/40)	Disintegrated, variable
Df(2R)robl-c	54B17-C4;54C1-4	(1/13)	Enlarged
Df(2R)BSC26	56C4;56D6-10	(2/11)	Suppressed
Df(2R)AA21	56F9-17;57D11-12	(7/14)	Disintegrated
Df(2R)59AD	59A1-3;59D1-4	(4/13)	Suppressed
Df(2R)Px2	60C5-6;60D9-10	(6/18)	Disintegrated
Df(2R)ED4065	60C8;60E8	(2/12)	Suppressed
Df(3L)Exel6087	62A2;62A6	(2/27)	Enlarged
Df(3L)BSC181	62A11;62B7	(7/28)	Disintegrated
Df(3L)HR119	63C2;63F7	(3/14)	Enlarged
Df(3L)pbl-X1	65F3;66B10	(3/10)	Suppressed
Df(3L)Scf-R6	66E1-6;66F1-6	(1/10)	Enlarged
Df(3L)vin5	68A2-3;69A1-3	(6/10)	Enlarged, variable
Df(3L)fz-GF3b	70C1-2;70D4-5	(6/17)	Disintegrated
Df(3L)81k19	73A3;74F	(3/7)	Suppressed
Df(3L)BSC8	74D3-75A1;75B2-5	(2/11)	Suppressed
Df(3L)ED4782	75F2;76A1	(3/13)	Disintegrated
Df(3L)rdgC-co2	77A1;77D1	(6/10)	Disintegrated, variable
Df(3L)ri-79c	77B-C;77F-78A	(5/18)	Suppressed
Df(3L)ri-XT1	77E2-4;78A2-4	(6/10)	Disintegrated, variable
Df(3L)ED4978	78D5;79A2	(5/10)	Small CZ
Df(3L)BSC223	79A3;79B3	(5/15)	Enlarged, variable
Df(3L)HD1	79D3-E1;79F3-6	(6/16)	Suppressed, disintegrated
Df(3R)Exel6144	83A6;83B6	(7/14)	Enlarged, disintegrated

Df(3R)3-4	82F3-4;82F10-11	(3/10)	Suppressed
Df(3R)ED5177	83B4;83B6	(5/15)	Enlarged
Df(3R)by10	85D8-12;85E7-F1	(2/11)	Enlarged
Df(3R)ry615	87B11-13;87E8-11	(5/12)	Enlarged, disintegrated
Df(3R)H-B79	92B3;92F13	(7/15)	Disintegrated
Df(3R)BSC43	92F7-93A1;93B3-6	(3/15)	Enlarged
Df(3R)e-N19	93B;94	(3/10)	Suppressed
Df(3R)e-R1	93B6-7;93D2	(9/18)	Disintegrated
Df(3R)Exel9012	94E9;94E13	(5/12)	Enlarged
Df(3R)BSC137	94F1;95A4	(5/15)	Suppressed
Df(3R)mbc-R1	95A5-7;95D6-11	(2/10)	Enlarged
Df(3R)crb-F89-4	95D7-D11;95F15	(4/13)	Suppressed
Df(3R)Exel6203	96E2;96E6	(5/11)	Disintegrated
Df(3R)Espl3	96F1;97B1	(10/21)	Enlarged, variable
Df(3R)IR16	97F1-2;98A	(5/15)	Enlarged, variable
Df(3R)BSC42	98B1-2;98B3-5	(3/12)	Suppressed
Df(3R)Dr-rv1	99A1-2;99B6-11	(5/13)	Enlarged CZ

Deficiencies with no modifying effects on Zfrp8/+ lymph gland phenotype

Df(2L)al, Df(2L)BSC107, Df(2L)Exel7011, Df(2L)dpp^{d14}, Df(2L)ED4651, Df(2L)JS17, Df(2L)BSC28, Df(2L)drm-P2, Df(2L)sc19-8, Df(2L)BSC165, Df(2L)ED250, Df(2L)BSC172, Df(2L)BSC109, Df(2L)Exel6011, Df(2L)BSC6, Df(2L)BSC7, Df(2L)XE-3801, Df(2L)BSC233, Df(2L)BSC41,Df(2L)Trf-C6R31, Df(2L)Exel7034, Df(2L)TE29Aa-11, Df(2L)ED611, Df(2L)N22-14, Df(2L)BSC240, Df(2L)BSC50, Df(2L)BSC143, Df(2L)BSC209, Df(2L)BSC50, Df(2L)J2, Df(2L)BSC214, Df(2L)BSC32, Df(2L)BSC241, Df(2L)BSC36, Df(2L)FCK-20, Df(2L)ED775, Df(2L)BSC252, Df(2L)ED793, Df(2L)ED3, Df(2L)TE35BC-24, Df(2L)H20, Df(2L)TW137, Df(2L)BSC148, Df(2L)Exel7070,

Df(2L)BSC256, Df(2L)Exel8038, Df(2L)BSC149, Df(2L)ED1203, Df(2L)pr-A16, Df(2L)BSC151, Df(2R)ED1484, Df(2R)ED1612, Df(2R)H3C1, Df(2R)ED1770, Df(2R)ED1791, Df(2R)Np5, Df(2R)w45-30n, Df(2R)B5, Df(2R)BSC298, Df(2R)X1, Df(2R)BSC281, Df(2R)ED2219, Df(2R)ED2247, Df(2R)BSC39, Df(2R)BSC199, Df(2R)CB21, Df(2R)BSC40, Df(2R)CX1, Df(2R)Exel7130, Df(2R)Exel7131, Df(2R)Jp1, Df(2R)ED2426, Df(2R)ED2436, Df(2R)ED2457, Df(2R)Jp8, Df(2R)Exel7144, Df(2R)Exel6064, Df(2R)BSC49, Df(2R)BSC161, Df(2R)BSC355, Df(2R)BSC45, Df(2R)14H10Y-53, Df(2R)BSC347, Df(2R)14H10W-35, Df(2R)P34, Df(2R)X58-12, Df(2R)BSC155, Df(2R)Kr10, Df(2R)ED50004, Df(3L)ED201, Df(3L)BSC362, Df(3L)ED4191, Df(3L)BSC289, Df(3L)Aprt-1, Df(3L)R-G7, Df(3L)ED4287, Df(3L)BSC119, Df(3L)Exel6092, Df(3L)ED4293, Df(3L)ED208, Df(3L)GN34, Df(3L)GN24, Df(3L)ZN47, Df(3L)BSC371, Df(3L)BSC410, Df(3L)BSC411, Df(3L)Exel6109, Df(3L)BSC27, Df(3L)BSC224, Df(3L)BSC117, Df(3L)ZP1, Df(3L)Exel6112, Df(3L)66C-G28, Df(3L)BSC389, Df(3L)h-i22, Df(3L)ED4421, Df(3L)BSC35, Df(3L)BSC283, Df(3L)ED4457, Df(3L)BSC14, Df(3L)ED4470, Df(3L)vin7, Df(3L)ED4486, Df(3L)BSC10, Df(3L)BSC12, Df(3L)ED4502, Df(3L)ED4543, Df(3L)fz-M21, Df(3L)brm11, Df(3L)st-f13,Df(3L)ED4606, Df(3L)Cat, Df(3L)BSC220, Df(3L)fz2, Df(3L)ED229, Df(3L)kto2, Df(3L)XS533, Df(3L)BSC451, Df(3L)Ten-m-AL29, Df(3L)ED230, Df(3L)BSC21, Df(3L)ED5017, Df(3L)1-16, Df(3R)e1025-14, Df(3R)ED5156, Df(3R)BSC464, Df(3R)Tpl10, Df(3R)WIN11, Df(3R)Scr, Df(3R)Antp17, Df(3R)ED7665, Df(3R)p712, Df(3R)BSC466, Df(3R)p-XT103, Df(3R)ED5330, Df(3R)BSC24, Df(3R)ED5428, Df(3R)Exel6155, Df(3R)M-Kx1, Tp(3;Y)ry506-85C, Df(3R)sbd105, Df(3R)P115, Df(3R)Cha7, Df(3R)Dl-BX12, Df(3R)ED5938, Df(3R)ED6025, Df(3R)Exel6272, Df(3R)ED10845, Df(3R)23D1, Df(3R)ED6096, Df(3R)BSC489, Df(3R)Exel6195, Df(3R)Exel9014, Df(3R)Exel6196, Df(3R)ED6220, Df(3R)BSC461, Df(3R)Exel6202, Df(3R)BSC321, Df(3R)ED6232, Df(3R)Tl-P, Df(3R)D605, Df(3R)BSC497, Df(3R)3450, Df(3R)L127, Df(3R)B81, Df(3 R)BSC502, Df(3R)Exel6214, Df(3R)BSC503, Df(3R)BSC504, Df(3R)Exel7378, Df(3R)ED6025

List of all additional deficiencies used in the screen that have weak, variable or no modifying effect of *Zfrp8*^{null}/+ lymph gland phenotype.
